# SHED-exosomes functionalized micro-nano bioactive glass/PCL membrane regulates macrophage polarization and promotes osteogenic differentiation

**DOI:** 10.1590/1678-7765-2025-0541

**Published:** 2026-01-30

**Authors:** Yongyong Yan, Haifeng Lan, Guohou Miao, Haiyan Wang, Zhengmao Li, Qing Zhang, Shuai Wang, Gang Wu, Richard T. Jaspers, Janak L. Pathak

**Affiliations:** 1 Guangzhou Medical University Guangdong Engineering Research Center of Oral Restoration and Reconstruction School and Hospital of Stomatology Guangzhou China Guangzhou Medical University, Guangdong Engineering Research Center of Oral Restoration and Reconstruction & Guangzhou Key Laboratory of Basic and Applied Research of Oral Regenerative Medicine, School and Hospital of Stomatology, Guangzhou, China; 2 Vrije Universiteit Amsterdam Faculty of Behavioural and Movement Sciences Department of Human Movement Sciences Amsterdam the Netherlands Vrije Universiteit Amsterdam, Amsterdam Movement Sciences, Faculty of Behavioural and Movement Sciences, Department of Human Movement Sciences, Laboratory for Myology, Amsterdam, the Netherlands.; 3 The Third Affiliated Hospital Guangzhou Medical University Department of Orthopaedic Surgery Guangzhou China The Third Affiliated Hospital of Guangzhou Medical University, Department of Orthopaedic Surgery, Guangzhou, China.; 4 Zunyi Medical University School and Hospital of Stomatology Zunyi Guizhou China Zunyi Medical University, School and Hospital of Stomatology, Zunyi, Guizhou 56300, China.; 5 Hangzhou Medical College Savid School of Stomatology Hangzhou China Hangzhou Medical College, Savid School of Stomatology, Hangzhou, 311399, China.

**Keywords:** Micro-nano bioactive glass, Exosomes, Stem cell, Osteogenic differentiation, Macrophage polarization

## Abstract

Micro-nano bioactive glass (MNBG) has bone regenerative potential. However, the difficulty of MNBG molding restricts its clinical applications in bone regeneration. Exosomes carry proteins, lipids, and nucleic acids for communication between cells. In this study, we adhered human exfoliated deciduous teeth (SHED)-derived exosomes (SHED-Exos) to electrospun micro-nano bioactive glass/polycaprolactone (MNBG/PCL) membrane to control inflammation and promote bone regeneration. MNBG/PCL membrane showed biocompatibility and osteogenic ability. Next, we demonstrated that SHED-Exos internalized into macrophages attenuated LPS-induced expression of M1-macrophage markers *iNOS*, *IL-6*, and *IL-1β* and upregulated M2-macrophage marker *IL-10* expression, indicating a switch from M1 to M2 macrophage phenotype. MNBG/PCL-loaded SHED-Exos membrane supported the survival and growth of mouse bone marrow stromal cells (mBMSCs). When M1 macrophages were co-cultured with mBMSCs on the membrane, the SHED-Exos-loaded membrane showed higher *Runx2*, *Alp*, and *Ocn* gene expression. Our findings indicate that SHED-Exos-functionalized MNBG/PCL membrane has anti-inflammatory and osteoinductive potential, suggesting its potential as a candidate material for bone regeneration.

## Introduction

The regeneration of bone defects remains a clinical challenge. The use of autologous and allogeneic bone grafting is a standard clinical therapy.^1^ However, autologous bone grafting can cause donor site damage, and allogeneic bone grafting risks rejection, resorption, and nonunion. Over the past few decades, numerous biomaterials have been rapidly developed as bone substitutes and show great potential for bone regeneration. Polycaprolactone (PCL) is commonly used as a biomaterial to repair bone defects, possessing suitable biodegradation time and bioabsorption properties for bone tissue regeneration.^2^ In addition to providing substantial mechanical support for regenerating bone tissue, electrospun PCL fibrous membranes mimic the extracellular matrix of tissues and regulate cell behavior.^3^ However, its application is limited due to its inferior bioactivity.

PCL’s surface exhibits poor wettability and forms weak interactions with biological fluids, hindering cell adhesion, proliferation, and differentiation. PCL scaffolds have previously been filled with bioactive inorganic fillers such as hydroxyapatite (HA), tricalcium phosphate (TCP), biphasic calcium phosphate (BCP), and bioactive glass (BG) to improve their bioactivity.^2,4-8^ BG is widely reported to participate in bone regeneration in guided tissue engineering materials.^9,10^ A variety of BG-containing biomaterials have been developed and commercialized.^9^ Micro-nano bioactive glass (MNBG) consists of uniformly sized BG particles at the micro-nanometer scale. MNBG has exhibited degradability,^11^ excellent bioactivity,^12^ and promotes osteogenesis and angiogenesis *in vitro*.^13^ Therefore, incorporating MNBG into PCL during scaffold preparation could complementarily promote bone repair.

In addition, implanted membranes may lead to inflammatory reactions and severe inflammation at the tissue interface during the surgical process, which also inhibits bone regeneration or promotes bone resorption.^14^ The interaction between macrophages and osteoblasts, a central focus of osteoimmunology, is crucial during bone regeneration.^15^ Macrophages are categorized into pro-inflammatory (M1) and anti-inflammatory (M2) phenotypes. Cells exhibiting the M1 phenotype, typically induced by bacterial lipopolysaccharide (LPS), secrete proinflammatory cytokines, including tumor necrosis factor-α (TNF-α), interleukin-1β (IL-1β), and interleukin-6 (IL-6). These cytokines can potentially inhibit the osteoblastic differentiation of stem cells and impede the successful integration of biomaterials. In contrast, M2 phenotype cells secrete anti-inflammatory cytokines such as interleukin-10 (IL-10) and transforming growth factor-beta (TGF-β), which are beneficial for immune regulation and tissue remodeling.^16^ Consequently, the development of immunomodulatory biomaterials must modulate the osteoimmune microenvironment toward an anti-inflammatory state.

Exosomes are a subtype of extracellular vesicles (EVs) 50–200 nm in diameter. Exosomes are secreted by various cells, including stem cells, B cells, dendritic cells, T cells, cancer cells, epidermal cells, and platelets, and are released into the extracellular milieu to exert biological effects. Exosomes comprise different bioactive molecules, including nucleic acids, lipids, enzymes, cytokines, and other proteins depending on their originating cells. Studies suggest that mesenchymal stem cell-derived exosomes (MSC-Exos) help decrease inflammation by facilitating the transition from M1 to M2 polarization and enhance the production of anti-inflammatory cytokines and chemokines. Compared to other types of MSCs, stem cells from human exfoliated deciduous teeth (SHED) offer many advantages, such as good proliferative potential, high cellular differentiation capacity, and the convenience of low or no pain. SHED are known to differentiate into bone-forming osteoblasts and have high immunomodulatory activity. A plethora of studies have revealed the regenerative potential of SHED in bone tissue engineering. However, whether SHED-derived exosomes (SHED-Exos) influence the osteoinductive ability of biomaterials in an osteoimmunity environment remains unclear.

This study aimed to generate an MNBG/PCL-loaded SHED-Exos (MNBG/PCL@Exos) membrane with osteogenesis-inducing capacity and immunomodulatory ability for guided bone regeneration. Our results indicate that the 1% MNBG/PCL membrane has good continuity, osteoinductive ability, and sustained exosome release ability. Moreover, the MNBG/PCL@Exos membrane supported the survival and growth of mouse bone marrow stromal cells (mBMSCs) and switched the phenotype of macrophages from M1 to M2, further improving the osteogenesis potential of mBMSCs co-cultured with M1 macrophages. Our results suggest that an anti-inflammatory and osteoinductive SHED-Exos functionalized MNBG/PCL membrane is a promising candidate, pending *in vivo* validation to augment osteogenesis.

## Methodology

### MNBG preparation and characterization

MNBG was prepared following previous work with some modifications.^17^ The process was as follows: equal volumes of deionized water and cyclohexane (30 mL) were mixed with 0.6 g of urea (Shanghai Macklin Biochemical Co., Ltd, Shanghai, China), 1 g of cetylpyridine bromide (CPB, Aladdin, Shanghai, China), and 0.92 g of isopropanol (Guangzhou Chemical Reagent Factory Co. Ltd, Guangzhou, China) under vigorous stirring at 70 °C to form a homogenous milky solution. Then, 2.7 mL of tetraethyl orthosilicate (TEOS, Guangzhou Chemical Reagent Factory Co. Ltd, Guangzhou, China) and 0.71 g of Ca (NO_3_)_2_·4H_2_O (Guangzhou Chemical Reagent Factory Co. Ltd, Guangzhou, China) were added to the solution and reacted for 16 h. The obtained products were collected by centrifugation, washed three times with absolute ethanol and deionized water, then freeze-dried (Christ, Alpha 1-4 LDPlus, Germany) for 48 h, and calcined (Nabertherm LT9/11/P330, Germany) at 600 °C for 5 h. To improve the calcium content of BG nanoparticles, the calcined powders were soaked in a Ca (NO_3_)_2_·4H_2_O/ethanol solution with a Si/Ca molar ratio of 4:1 for 12 h. The mixture solution was stirred until the ethanol was completely volatilized, and the obtained complexes were calcined at 650 °C for 3 h to obtain MNBG nanoparticles. A scanning electron microscope (SEM, Merlin, Germany) was used to characterize the morphology and structure of the MNBG nanoparticles.

### Fabrication of MNBG/PCL membrane

MNBG nanoparticles at 0.5% or 1% (w/w) concentration were added to 1,1,1,3,3,3-hexafluoro-2-propanol (HFIP, Aladdin, Shanghai, China) solution and dispersed by ultrasonic treatment for 30 min. Then, 8% (w/w) PCL (Aladdin, Shanghai, China) was added to the mixture solution and intensely stirred for 4 h until a homogeneous solution was obtained. The solution was loaded into a 10 mL syringe connected to a 17 gauge metallic needle by a Teflon tube and fed at a flow rate of 1 mL/h using the Nanon electrospinning setup (MECC, Japan). A voltage of 20 kV was chosen to produce a stable jet. The distance between the needle and the plate collector was 15 cm. The membrane was collected on an aluminum foil and stripped down for further study. Fiber morphology of the membranes was imaged using SEM (S3400N, Japan). Energy Dispersive Spectroscopy (EDS, Hitachi, Japan) was used to determine the membrane content of calcium and silicon.

### Mechanical properties assessments

Before the tensile test, MNBG/PCL nanofibrous matrices were cut into a dumbbell shape according to the Type 2 sample in GB/t 528-2009, with a length of 25 mm, a width of 4 mm, and a thickness ranging from 0.20 to 0.30 mm. Briefly, the membranes were pulled at a rate of 10 mm/min until the sample broke at 25 °C with a relative humidity of 20%. The mechanical performance test was performed using an Instron 3366 (Instron, USA) universal tensile tester to obtain stress-strain curves of the fibrous matrices.

### Characterization of 8%PCL and MNBG/PCL membrane

The 8% PCL and MNBG/PCL membranes were analyzed using an X-ray diffractometer (XRD) (D8 Advance, Bruker, Germany). The chemical structure of samples was analyzed by attenuated total reflectance-Fourier transform infrared spectroscopy (Scientific iN10, Thermo, USA). The water contact angle (WCA) of the nanoﬁbrous membranes was measured by a video contact angle instrument (KRUSS, DSA25, Germany).

### Cell culture

The mBMSCs were purchased from Cyagen Biosciences Technology (MUBMX-01001, Guangzhou, China) and cultured in Dulbecco’s modified Eagle’s medium (DMEM; Gibco, MA, USA) containing 10% fetal bovine serum (FBS; Gibco, MA, USA) and 1% penicillin/streptomycin (Gibco, MA, USA). For osteogenic differentiation studies, mBMSCs were cultured in osteogenic induction medium (OM) consisting of complete medium supplemented with 50 μg/mL L-ascorbic acid (Sigma Aldrich, St. Louis, MO, USA), 10 mM β-glycerophosphate (Sigma Aldrich, St. Louis, MO, USA), and 10 nM dexamethasone (Sigma Aldrich, St. Louis, MO, USA). RAW264.7 murine macrophages, purchased from Shanghai Cell Bank (Shanghai, China), were cultured in DMEM containing 10% FBS and 1% penicillin/streptomycin.

SHED were isolated from healthy children’s deciduous teeth (6–12 years old) after obtaining informed consent from the parents. This study was approved by the Medical Ethics Committee of the Affiliated Stomatology Hospital of Guangzhou Medical University (KY2019005). The SHED isolation method was described previously.^18^ SHED were expanded using an alpha modification of Eagle’s medium (α‐MEM, Gibco, MA, USA) with 10% FBS and 1% penicillin/streptomycin.

mBMSCs, RAW264.7, and SHED were cultured under standard conditions (37 °C, 5% CO₂). Cells were passaged when reaching 80–90% confluency. SHED passages 3 to 6 were used for all subsequent assays.

### Flow cytometry analysis

SHED of the third passage were washed with PBS and exposed to 0.25% trypsin-EDTA (Thermo Fisher Scientific, Waltham, MA, USA) for 1–2 min. Then, cells were suspended in PBS, counted, diluted to 0.5 × 10^6^ cells/mL, transferred into special flow cytometer tubes, and incubated with specific fluorochrome-conjugated antibodies on ice for 30 min. Next, cells were washed with PBS, centrifuged at 400 g for 5 min, and resuspended in PBS. Isotype controls were prepared. The BD FACSAria™ IIU flow cytometer and BD FACSDiva software (BD Biosciences, San Jose, CA, USA) were used to analyze the cell samples. Flow cytometry data were presented as a percentage of cells having specific cell surface markers for cell identification and as mean fluorescence intensity (MFI) for other flow measurements. The antibodies used to evaluate SHED surface markers were: CD73 (4ABIO, Beijing, China), CD90, CD105, CD19, CD45, HLA-DR (BioLegend, San Diego, CA, USA), and CD34(BD Biosciences, San Jose, CA, USA).

### Isolation, purification, and identification of exosomes

SHED of passage (P) 4–P6 were cultured in medium without FBS for 48 h to isolate exosomes. The medium was collected, and exosomes were extracted using the ultracentrifugation method. The supernatant was centrifuged at 2000 g for 20 min and 10,000 g for 40 min at 4 °C to remove cells and cellular debris. Then, the supernatant was ultracentrifuged (Ultracentrifuge, Beckman Coulter, USA) at 100,000 g for 70 min to collect the exosome pellet and additionally washed with phosphate-buffered saline (PBS, Gibco, MA, USA) at 100,000 g for 70 min to eliminate protein contamination. Nanoparticle tracking analysis (NTA, Nanosight LM10 system, Amesbury, UK) was used to identify the particle size, concentration, and size distribution.

### Transmission electron microscopy

Exosomes were fixed in 2% paraformaldehyde, washed, and loaded onto formvar-carbon-coated grids. After washing, they were postfixed in 2% glutaraldehyde for 2 min, washed, and contrasted in 2% phosphotungstic acid for 5 min. Samples were washed, dried, and examined by transmission electron microscopy (TEM, HT7700 Hitachi, Japan) to assess morphological characteristics.

### Western blot analysis

The expression of exosome protein markers, including CD9 (ab92726, Abcam, United Kingdom), CD63 (ab217345, Abcam, United Kingdom), CD81 (ab109201, Abcam, United Kingdom), and TSG101 (ab125011, Abcam, United Kingdom), was analyzed by western blotting. Western blot analysis was performed as follows: exosome proteins were extracted using the Membrane and Cytosol Protein Extraction Kit (Beyotime, Shanghai, China), and their concentrations were quantified using the Bicinchoninic Acid Protein Assay Kit (Beyotime, Shanghai, China). Subsequently, samples were electrophoresed on 10% sodium dodecyl sulfate-polyacrylamide gels, transferred to Immobilon membranes (Millipore, Bedford, MA, USA), and then incubated in QuickBlock™ Blocking Buffer (Beyotime, Shanghai, China) for 15 min. Then, using standard techniques, the membranes were probed with antibodies, including CD9, CD63, CD81, and TSG 101. The membranes were incubated with secondary antibodies (Affinity) at room temperature for 1 h. Labeling was visualized using the Affinity ECL Kit (Affinity Biosciences, Cincinnati, USA) and the gel-view system (BLT Photon Technology, China).

### Exosomes uptake assay

Exosomes were labeled with PKH-26 (Sigma-Aldrich, St. Louis, MO, USA) to determine their uptake by RAW264.7 cells. Exosomes were diluted in 1 mL Diluent C, and 4 µL PKH26 dye diluted in 1 mL Diluent C were incubated together. After 4 min, 2 mL 0.5% BSA/PBS was added to bind the excess dye for 5 min. Then, labeled exosomes were washed in PBS at 200,000 g for 1 h. Subsequently, the labeled exosomes were co-cultured with RAW264.7 cells for 4 h. Following the incubation, cells were washed twice with PBS and stained with Hoechst (Sigma-Aldrich, St. Louis, MO, USA) for 5 min. Images were captured with a Leica TCS-SP8 confocal imaging system (LEICA, Germany).

### Exosomes loading and release assay on MNBG/PCL membrane

Exosomes were physically attached to the surface of the MNBG/PCL membrane. The 1% MNBG/PCL membrane was immersed in 10 μg/mL PKH26-labeled exosome solution for 12 h at 4 °C. Images were captured with a Leica TCS-SP8 confocal imaging system (LEICA, Germany) to assess the distribution of exosomes on the MNBG/PCL membrane. The release of exosomes from the MNBG/PCL membrane was evaluated in PBS. The MNBG/PCL membranes loaded with PKH26-labeled exosomes were incubated in PBS at 37 °C. At designated time points (1 d, 2 d, 3 d, and 4 d) (n=4 per time point), the PBS was collected and replaced with fresh PBS. Exosome release was quantified by measuring the fluorescence intensity of the collected PBS using a fluorescence microplate reader (TECAN Infinite 200 PRO, Austria).

### Immunomodulatory properties of SHED-Exos and MNBG/PCL@Exos membrane

Lipopolysaccharide (LPS) was used to induce inflammation in M1 macrophage polarization. RAW264.7 cells (1×10^5^/well) were seeded into a 6-well plate and treated with 1 μg/mL E. coli LPS (Sigma Aldrich, Shanghai, China) for 4 h. To analyze whether SHED-Exos could regulate immune function, two doses of SHED-Exos (5 and 10 µg/mL) were incubated with M1 phenotype macrophages. After 24 h, total cell RNA was collected after which RT-qPCR was performed to assess gene of M1 phenotype markers (*iNOS, IL-6* and *IL-1β*) and M2 phenotype markers (*CD206* and *IL-10* ).

### Real-time quantitative PCR (RT-qPCR) analysis

RT-qPCR was used to detect the expression of osteoblastic differentiation markers: runt-related transcription factor 2 (*Runx2*), alkaline phosphatase (*Alp*), and osteocalcin (*OCN*); M1 macrophage markers: inducible NOS (*iNOS*), interleukin-6 (*IL-6*), and interleukin-1 beta (*IL-1β*); and M2 macrophage markers: interleukin-10 (*IL-10*) and *CD206*. Cells were seeded in 6-well plates at 100,000 cells/well density. After various treatments, total RNA was extracted from cells using the SteadyPure Universal RNA Extraction Kit (Accurate Biology, China) and reverse transcribed into cDNA using a PrimeScript RT reagent kit with gDNA Eraser (Takara Biotechnology, Japan) following manufacturer’s protocols. RT-qPCR was performed using the TB Green Pre-mix Ex Taq II kit (Takara Biotechnology, Japan). Table 1 lists the primer sequences. Glyceraldehyde 3-phosphate dehydrogenase (GAPDH) was used as a reference gene. The relative level of mRNA expression was defined using the 2^-^ method. Relative mRNA expression levels were standardized to the level of the reference gene GAPDH. Each reaction was performed in triplicate.

**Table 1:** Primers used for RT-qPCR analysis.

Gene	Acc No.	Primer Sequence (5'->3')	Product Length (bp)
*Mus-Gapdh*	NM_001411843.1	F: TGTGTCCGTCGTGGATCTG	150
		R: TTGCTGTTGAAGTCGCAGGA	
*Mus-Alp*	NM_007431.3	F: TGCCTACTTGTGTGGCGTGAA	164
		R: TCACCCGAGTGGTAGTCACAATG	
*Mus-Ocn*	NM_007541.3	F: AGCAGCTTGGCCCAGACCTA	178
		R: TAGCGCCGGAGTCTGTTCACTAC	
*Mus-Runx2*	NM_001271630.2	F: CACTGGCGGTGCAACAAGA	144
		R: TTTCATAACAGCGGAGGCATTTC	
*Mus-IL-6*	NM_031168.2	F: ATAGTCCTTCCTACCCCAATTTCC	93
		R: GATGAATTGGATGGTCTTGGTCC	
*Mus-IL-1β*	NM_008361.4	F: TGGAGAGTGTGGATCCCAAG	128
		R: GGTGCTGATGTACCAGTTGG	
*Mus-IL-10*	NM_010548.2	F: GGTTGCCAAGCCTTATCGGA	191
		R: ACCTGCTCCACTGCCTTGCT	
*Mus-CD206*	NM_008625.2	F: GGCTGATTACGAGCAGTGGA	184
		R: ATGCCAGGGTCACCTTTCAG	
*Mus-iNOS*	NM_010927.4	F: AAACCCCTTGTGCTGTTCTC	287
		R: GTCTCTGGGTCCTCTGGTCA	

### Alizarin red staining

MNBG/PCL membranes were placed on a 48-well plate and soaked in medium containing 10% FBS overnight. mBMSCs (2.5×10^4^/well) were cultured with osteogenic induction medium for 28 days.^19,20^ Cells were washed three times with phosphate-buffered saline (PBS), fixed with 4% paraformaldehyde for 30 min, and stained with 1% alizarin red S staining solution (pH 4.2, Sigma-Aldrich) at room temperature. Images were captured using a Leica EZ4-HD imaging system (LEICA, Germany).

### Cell viability on MNBG/PCL@Exos membrane

MNBG/PCL membranes were placed on a 48-well plate and soaked in medium containing 10% FBS overnight. In total, three doses (0, 5, and 10 µg/mL) of exosomes were immersed for 12 h at 4 °C. mBMSCs (2.5×10^4^/well) were added and incubated for 1 or 3 days. Each group of cells was stained with Calcein AM (10 μL) and propidium iodide (PI) (15 μL) (Calcein AM /PI Double Stain Kit, Solarbio) at 37 °C for 15 min. The samples were visualized using a Leica TCS-SP8 confocal 3D imaging system (LEICA, Germany). Calcein AM was used to stain live cells (which emitted green fluorescence), while red fluorescence was obtained from dead cells due to PI staining.

### Cell viability assay (CCK-8)

The proliferation and viability of mBMSCs cultured on different membranes were evaluated using the Cell Counting Kit-8 (CCK-8, Dojindo, Japan). Briefly, mBMSCs (passages 3–5) were seeded onto MNBG/PCL membranes placed in 24-well plates at a density of 1×10⁴ cells per well. After incubation for 24 h and 72 h, the culture medium was replaced with 400 μL of fresh DMEM containing 10% CCK-8 reagent and incubated at 37 °C for 2 h. Subsequently, 100 μL of the supernatant was transferred to a 96-well plate, and the absorbance was measured at 450 nm using a microplate reader (Bio-Rad, USA). Cell viability was expressed as the relative optical density (OD) value compared to the control group without exosomes.

### Statistical analysis

Data were analyzed using SPSS software (Chicago, IL, USA). Comparisons between two groups were analyzed by independent two-tailed Student’s t-tests. Comparisons between more than two groups were analyzed by one-way ANOVA followed by Tukey’s post hoc test. The results were expressed as the mean ± standard deviation (SD). For all tests, statistical significance was accepted for *p*-values lower than 0.05.

## Results

### Preparation, characterization, and mineralization of MNBG/PCL membrane

**Figure 1:**
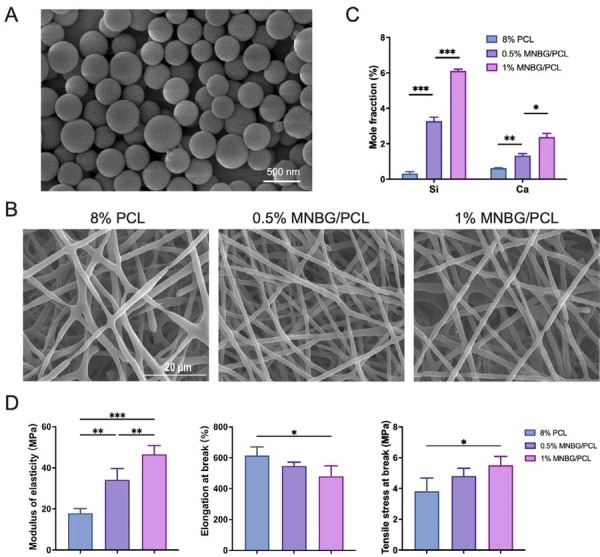
MNBG/PCL membrane preparation, characterization, and osteogenic properties. (A) SEM images showing the surface topography and characterization of bioactive micro-nano glass nanoparticles (MNBG). (B) SEM images of the electrospun nanofibrous membranes. (C) Comparison of calcium and silicon content in the MNBG/polycaprolactone (PCL) membrane. (D) Mechanical characterization results (modulus of elasticity, elongation at break, and tensile stress at break). Data are presented as mean ±SD, n=4. Significant difference compared to the PCL group *p<0.05, **p<0.01, and ***p<0.001.

MNBG particles were effectively created through a sol-gel process paired with gelation-induced phase separation technology, utilizing CPB as a template. SEM analysis revealed that the particles were spherical, ranging from 200 to 400 nm, and the surface had nanopore sizes beneficial to cell adhesion (Figure 1A). The composite scaffolds (8% PCL with 0%, 0.5%, and 1% BG, respectively) were prepared by electrospinning, and SEM was used to determine the structure. As shown in Figure 1B, the membranes featured a fibrous morphology. MNBG particles were well dispersed in the PCL matrix and did not affect the continuity of the PCL fibers. The presence of calcium (Ca) and silicon (Si) in the MNBG/PCL membrane was semi-quantified by EDS (Figure 1C). As the MNBG content rose, the levels of Ca and Si also increased.

**Figure 2:**
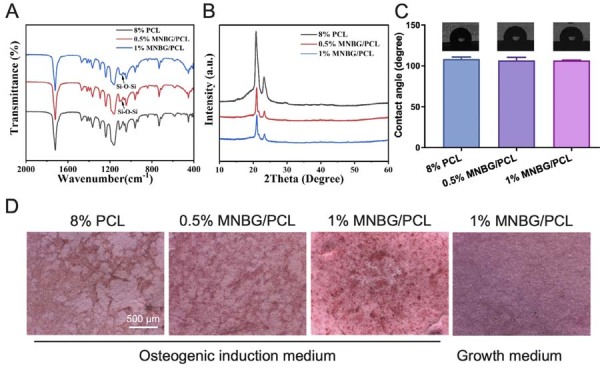
MNBG/PCL membrane characterization. (A) Fourier transform infrared spectroscopy (FTIR) analysis showing peaks around 1090 cm −1. (B) X-ray diffraction (XRD) pattern of the membranes. (C) Contact angle analysis of the membranes. (D) Alizarin red staining of mBMSCs on the MNBG/PCL membrane after 28 days of osteogenic differentiation induction.

The mechanical properties of the fabricated membranes—modulus of elasticity, elongation at break, and tensile stress at break—were assessed under uniaxial tensile loading (Figure 1D). In general, incorporating MNBG led to increased stiffness of mechanical properties. Our results indicate that the elastic modulus of the 1% MNBG/PCL membrane was 2.5-fold higher compared to the pure 8% PCL membrane, the tensile stress at break was 1.4 times higher, while the elongation at break was 22% lower. FTIR (Figure 2A) and XRD (Figure 2B) were used to analyze the chemical composition of the engineered membranes. FTIR confirmed the presence of MNBG in the MNBG/PCL membrane, indicated by an enhanced peak attributed to Si-O stretching vibration (1090 cm^1^). XRD spectra showed that diffraction peaks appeared at 2θ=21.2° and 23.7°, corresponding to those of PCL crystal’s (110) and (220) crystal faces, respectively. The intensity of the diffraction peaks decreased with the addition of bioactive glass, indicating a reduction in the crystallinity of polycaprolactone. The diffraction peak of bioactive glass was not evident due to its amorphous structure. Additionally, contact angle measurements of the membrane material indicated that incorporating MNBG does not lead to significant changes in the contact angle (Figure 2C). This could be attributed to the hydrophilic MNBG being embedded within the PCL fibers.

After mBMSCs were incubated on the MNBG/PCL membrane with osteogenic induction medium for 28 days, the 1% MNBG/PCL membrane showed a more significant impact on matrix mineralization in mBMSCs culture than both the 8% PCL and 0.5% MNBG/PCL membranes (Figure 2D). This was probably caused by the Ca and Si originating from MNBG. We chose the 1% MNBG/PCL membrane for the following experiments.

### Identification of SHED-derived exosomes

**Figure 3:**
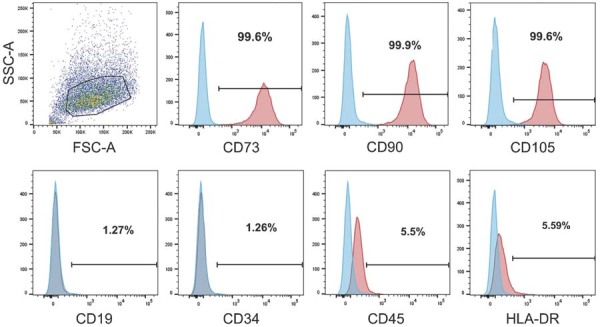
Expression of cell surface markers on SHED cells as determined by flow cytometry.

**Figure 4:**
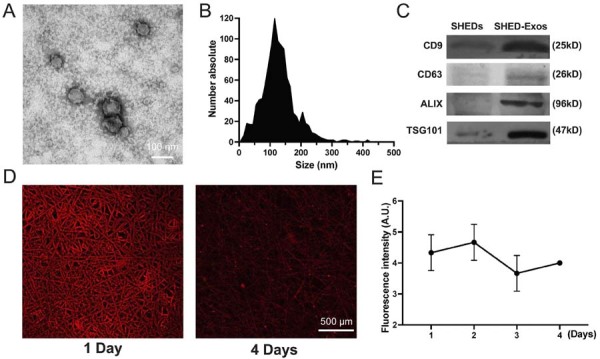
Characterization of exosomes derived from SHED cells. (A) Morphology of exosomes observed by transmission electron microscopy (TEM). (B) Particle size distribution of exosomes measured by NanoSight analysis: the mean size ± SD of exosomes was 119 ± 42 nm. (C) Western blot analysis of the exosomal surface markers. (D) Confocal microscopy showing exosomes adhered to the MNBG/PCL membrane. (E) Fluorescence microplate reader detection of exosomes released in PBS from MNBG/PCL nanofiber membranes.

We successfully isolated SHED with typical characteristics (Figure 3). Subsequently, we used conventional ultra-centrifugation to separate exosomes from the SHED culture medium. SHED-derived exosomes (SHED-Exos) were analyzed using transmission electron microscopy (Figure 4A). The obtained vesicles ranged from 50 to 200 nm in size (Figure 4B). Exosome characterization was extended by detecting exosomal proteins in SHED-Exos, which included Alix, TSG101, CD63, and CD9. Western blot analysis confirmed these exosomal marker proteins on SHED-Exos (Figure 4C). Laser confocal scanning microscope images revealed that the PKH26-labeled exosomes (red dots) were evenly spread across the membrane surface following immobilization (Figure 4D). The fluorescence intensity plate reader detected daily changes in the PBS supernatant and the supernatant fluorescence intensity. Exosomes were shown to be released from the membrane for up to 4 days (Figure 4E).

### SHED-Exos and SHED-Exos-loaded MNBG/PCL membranes regulate macrophage immunomodulation

**Figure 5:**
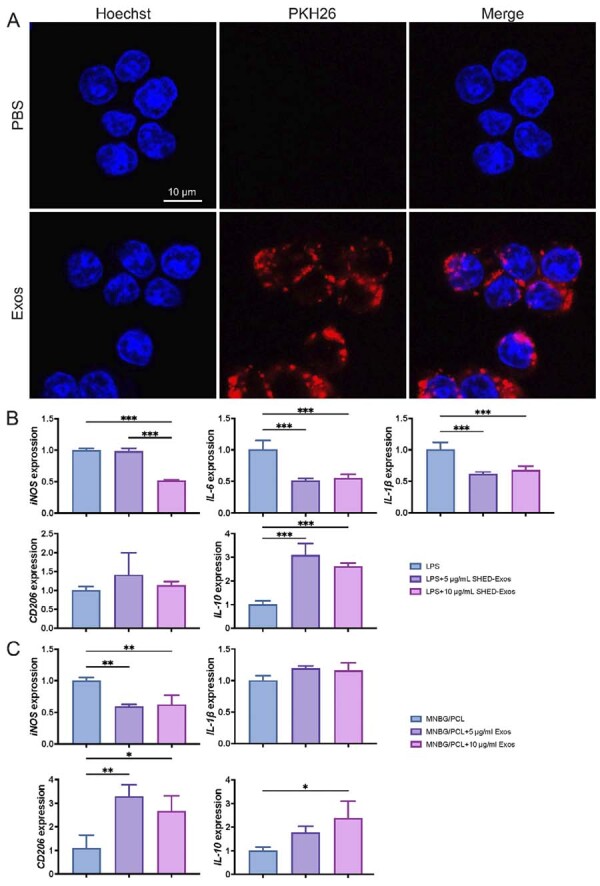
Induction of macrophages switching from M1 to M2 by SHED-Exos and the MNBG/PCL@Exos membrane. (A) Immunofluorescence analysis showing the uptake of SHED-Exos by macrophages. Red indicates SHED-Exos, and blue indicates nuclei. (B) mRNA expression levels of M1 phenotype markers *iNOS*, *IL-6*, and *IL-1β* in macrophages relative to Gapdh. (C) mRNA expression of M2 phenotype markers *CD206* and *IL-10* in macrophages. (D) mRNA expression levels of M1 phenotype markers (*iNOS* and *IL-1β*) and M2 phenotype markers (*CD206* and *IL-10*) in M1 phenotype macrophages cultured on the MNBG/PCL@Exos membrane for 24 h. Data are presented as the mean±SD, n=3, *p<0.05, **p<0.01, and ***p<0.001.

We found that macrophages took up PKH26-labeled SHED-Exos within 4 h (Figure 5A). To evaluate whether SHED-Exos treatment can convert macrophages from M1 to M2, we cultured LPS-induced M1 macrophages with SHED-Exos. We measured the mRNA expression levels of M1 markers (*iNOS, IL-6*, and *IL-1β*) and M2 markers (*CD206, IL-10*) using various exosome concentrations. SHED-Exos (10 μg/mL) significantly downregulated the expression of *iNOS, IL-6*, and *IL-1β* in M1 macrophages, while upregulating *IL-10* (Figure 5B). These results suggest that SHED-Exos aids in the transition of macrophages from the M1 to the M2 phenotype.

To further confirm the immunomodulatory effect of SHED-Exos-loaded MNBG/PCL membranes, M1 phenotype macrophages were seeded on the membranes and incubated for 24 h. The data showed that the SHED-Exos-loaded membrane could downregulate *iNOS* mRNA expression and upregulate *CD206* and *IL-10* mRNA expression levels compared with the pristine MNBG/PCL membrane, though IL-1β expression levels did not show a significant difference (Figure 5C). Our results indicate that SHED-Exos and MNBG/PCL@Exos membranes could switch the macrophage phenotype from M1 to M2.

### Biocompatibility and osteogenesis of mBMSCs on MNBG/PCL@Exos membrane

**Figure 6:**
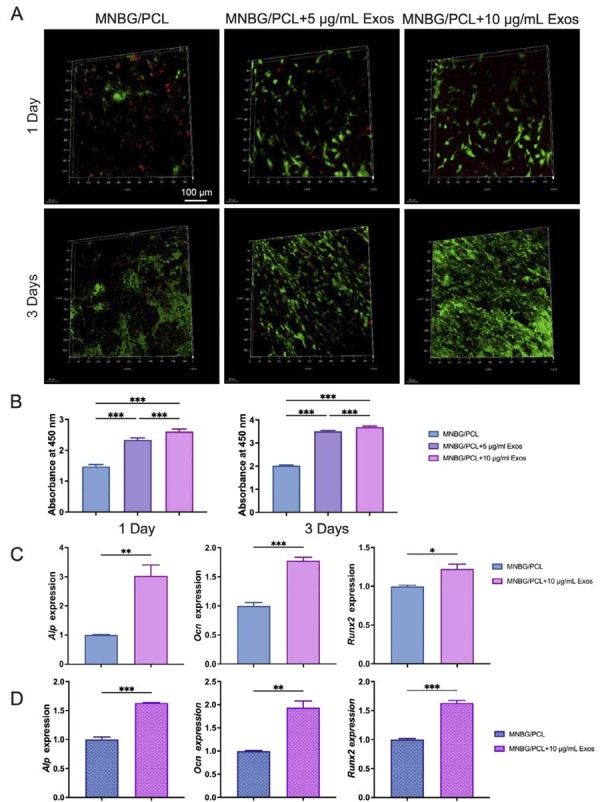
Biocompatibility and osteogenesis of mBMSCs on the MNBG/PCL@Exos membrane. (A) Live/dead staining of mBMSCs cultured on the MNBG/PCL@Exos membrane surface for 1 or 3 days. Green represents living cells, and red represents dead cells. (B) Cell viability of mBMSCs cultured on the MNBG/PCL membranes containing various concentrations of SHED-Exos for 1 and 3 days, measured by CCK-8 assay at 450 nm. (C) Expression of osteogenesis-related genes (*Alp*, *Ocn*, and *Runx2*) after pure mBMSCs were osteogenically induced and cultured on the membrane’s surface for 4 days. (D) Expression of osteogenesis-related genes (*Alp*, *Ocn*, and *Runx2*) after mBMSCs and M1 macrophages (9:1) were co-cultured on the membrane surface and osteogenically induced for 4 days. Data are presented as the mean±SD, n=3, * p<0.05, **p<0.01, and ***p<0.001.

The attachment and viability of osteogenic precursor cells on the membrane surface are critical for effective osteointegration. The attachment and viability of mBMSCs on the membranes were assessed using live-dead fluorescence staining. The images showed higher adhesion and survival of mBMSCs on SHED-Exos-coated MNBG/PCL membranes (Figure 6A). Moreover, 3-day incubation showed increased live cell density over time on the SHED-Exos-coated MNBG/PCL membranes (Figure 6A). CCK-8 assay results (Figure 6B) show that SHED-Exos effectively promoted the proliferation and metabolic activity of BMSCs compared with the MNBG/PCL control. These results were consistent with live/dead cell staining results, suggesting that MNBG/PCL@Exos membranes possessed favorable biocompatibility and were appropriate for further functional assessments.

The impact of SHED-Exos-coated MNBG/PCL membranes on mBMSCs osteogenic differentiation was examined by culturing mBMSCs under osteogenic induction on the SHED-Exos-coated MNBG/PCL membrane surface for 4 days. *Alp, Ocn, and Runx2* expression was significantly upregulated on the SHED-Exos-coated MNBG/PCL membrane compared with the MNBG/PCL membranes (Figure 6C). To assess osteogenic potential in an inflammatory immune microenvironment, mBMSCs were co-cultured with M1 macrophages (9:1) on the SHED-Exos-coated MNBG/PCL membrane surface, and osteogenesis-related gene expression was quantified on day 4. A notable enhancement of *Alp, Ocn*, and *Runx2* expression was observed in the SHED-Exos-coated MNBG/PCL group compared with the MNBG/PCL group (Figure 6D). These results indicate that the SHED-Exos-coated MNBG/PCL membrane can promote osteogenic differentiation of precursor cells in physiological and inflammatory immune microenvironments.

## Discussion

Compromised osteogenesis and inflammatory reactions are critical factors affecting bone tissue engineering.^21,22^ M1 to M2 macrophage polarization has shown promising bone regenerative effects.^23-25^ Our designed SHED-Exos-functionalized MNBG/PCL biomembrane promoted M1 to M2 macrophage polarization and osteogenic differentiation of precursor cells in physiological and inflammatory immune microenvironments. Our results showed that this hybrid scaffold effectively promotes macrophage polarization toward the M2 phenotype and enhances osteogenic differentiation of mBMSCs under both physiological and inflammatory conditions.

All implanted biomaterials cause foreign body responses, such as the degradation of biomaterials, which might elicit a robust inflammatory response, inhibiting bone regeneration.^26^ Macrophages, as indispensable immune cells, are extensively studied for osteoimmunomodulation.^27^ Controlling of macrophage plasticity, ranging from M1 to M2 phenotypes, is a critical process in the immunomodulatory control of bone regeneration.^28^ M1 macrophages can secrete pro-inflammatory cytokines, such as iNOS, IL-6, IL-1β, TNF-α, IL-12, IL-18, IL-23, CCL2, CXCL1-3, CXCL5, and CXCL8-10.^29^ M2 macrophages can release high levels of TGF-β, IL-10, IL-4, IL-13, CD206, CD163, and CCR2.^30^ M2 macrophages perform anti-inflammatory, tissue regeneration, and repair effects. MSC-Exos has been reported to alleviate inflammation by inhibiting macrophage M1 polarization and promoting M2 polarization.^31^ This study demonstrated that SHED-Exos could internalize into macrophages and mitigate inflammatory responses. The SHED-Exos-loaded MNBG/PCL membrane significantly downregulated the M1-related marker (*iNOS*) and upregulated M2-related markers (*CD206* and *IL-10*) in LPS-induced M1 macrophages. Our study indicated that the SHED-Exos-loaded MNBG/PCL membrane could induce M1 to M2 macrophage phenotype switching. Moreover, studies have demonstrated that MSC-Exos-induced M2 polarization can persist for several days following exosome administration,^32^ indicating that the M2 phenotype may remain stable even after SHED-Exos are withdrawn. In conjunction with our SHED-Exos release experiments using membranes, these findings underscore the ability of SHED-Exos to modulate macrophage behavior and establish a favorable osteoimmune microenvironment.

The adhesion and survival of osteogenic and angiogenic precursor cells on the biomaterial surface are vital for successful bone regeneration.^22^ The hydrophobic nature of PCL is averse to cell attachment and proliferation, which remains a major challenge.^33^ Inorganic biomaterials, such as MNBG, have been incorporated into PCL to enhance cell adhesion and proliferation.^7^ In our study, XRD analysis indicated a slight reduction in PCL crystallinity after MNBG incorporation, suggesting that MNBG might slightly accelerate PCL degradation through increased hydrophilicity. This agrees with previous findings that MNBG/PCL composites enhance hydrolytic degradation and ion release while maintaining structural integrity.^34^ Moreover, released Ca and Si ions from MNBG are known to stimulate osteogenic differentiation and may synergize with SHED-Exos to promote matrix mineralization.

The MNBG/PCL@Exos membrane exhibited a sustained and smooth release of SHED-Exos over four days, a release profile characteristic of physisorbed exosome systems. Although the detected release duration is relatively short compared to the overall bone healing process, it precisely coincides with the early inflammatory phase (the first 3–5 days after injury), during which immune regulation is most critical. Moreover, the integrity of exosomes on the MNBG/PCL membrane beyond four days remains questionable. Therefore, the current design is strategically aimed at delivering timely immunomodulatory signals rather than achieving prolonged release. Future studies will focus on enhancing exosome retention through surface modification strategies, such as polydopamine (PDA)^35^ coatings—approaches that have been shown to improve exosome adhesion and extend release duration to 7–14 days. More importantly, demonstrating the complete preservation and controlled release of SHED-Exos from the MNBG/PCL scaffold via integrated quantitative methods—such as nanoparticle tracking analysis (NTA) or ELISA for exosomal markers (CD63/CD9)—will be a key advancement in future plans.

**Figure 7:**
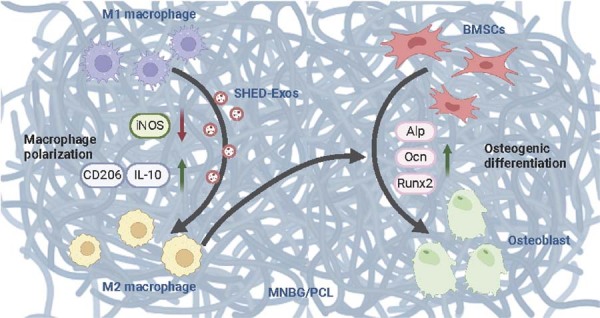
Schematic diagram of the SHED-Exos-loaded MNBG/PCL membrane regulating macrophage polarization to mediate osteogenesis. The integration of SHED-Exos with the MNBG/PCL membrane provides a dual function: It not only serves as a scaffold for bone tissue engineering but also actively participates in the modulation of the immune response, which is crucial for successful bone healing.

Osteogenesis is the most fundamental process of bone tissue regeneration. The SHED-Exos-loaded MNBG/PCL membrane induced greater osteogenic differentiation of BMSCs than the MNBG/PCL membrane. Furthermore, osteogenesis is profoundly impacted by the immunomodulatory properties of biomaterials. Effective bone regeneration depends on the careful coordination of inflammatory and bone-forming cells. Growing evidence supports that M2 macrophages are more favorable for osteogenesis, especially in the context of biomaterial implantation.^36,37^ Bone graft biomaterials that can provide anabolic crosstalk between macrophages and MSCs are in high demand. We directly co-cultured BMSCs and M1 macrophages on MNBG/PCL or SHED-Exos-loaded MNBG/PCL membranes. The results revealed that the SHED-Exos-loaded MNBG/PCL membrane promoted the expression of osteogenic differentiation markers compared with the MNBG/PCL membrane. These results confirmed that the SHED-Exos-loaded MNBG/PCL membrane can potentially promote osteogenic differentiation of precursor cells even under an inflammatory immune microenvironment (Figure 7).

However, this study has several limitations. First, only cytokine mRNA levels were measured; subsequent research should include techniques such as ELISA and miRNA analysis to confirm M1 and M2 cytokines at the protein level. Secondly, we did not evaluate the structural integrity of exosomes after release on the MNBG/PCL membrane. Nonetheless, the observed bioactivity suggests that SHED-Exos maintain their functional stability throughout the delivery and immobilization process. Finally, *in vivo* validation is required to verify long-term biocompatibility, degradation patterns, and regenerative activity.

## Conclusions

In conclusion, SHED-Exos and SHED-Exos-loaded MNBG/PCL membranes displayed the potential for M1 to M2 macrophage phenotype switching. Compared to MNBG/PCL nanofibers, SHED-Exos-loaded MNBG/PCL membranes exhibited direct osteoinductive properties in both basal physiological and inflammatory environments. Our findings demonstrate the immunomodulatory and osteoinductive properties of SHED-Exos-functionalized MNBG/PCL membranes *in vitro*, highlighting their potential as a promising candidate for bone regeneration, pending further *in vivo* validation.

## References

[1] Higgins TF, Marchand LS (2018). Basic science and clinical application of reamed sources for autogenous bone graft Harvest. J Am Acad Orthop Surg.

[2] Murugan S, Parcha SR (2021). Fabrication techniques involved in developing the composite scaffolds PCL/HA nanoparticles for bone tissue engineering applications. J Mater Sci Mater Med.

[3] Banimohamad-Shotorbani B, Rahmani Del Bakhshayesh A, Mehdipour A, Jarolmasjed S, Shafaei H (2021). The efficiency of PCL/HAp electrospun nanofibers in bone regeneration: a review. J Med Eng Technol.

[4] Poh PSP, Hutmacher DW, Holzapfel BM, Solanki AK, Stevens MM, Woodruff MA (2016). In vitro and in vivo bone formation potential of surface calcium phosphate-coated polycaprolactone and polycaprolactone/bioactive glass composite scaffolds. Acta Biomater.

[5] Li X, Yin HM, Luo E, Zhu S, Wang P, Zhang Z (2019). Accelerating bone healing by decorating BMP-2 on porous composite Scaffolds. ACS Appl Bio Mater.

[6] Helaehil JV, Lourenço CB, Huang B, Helaehil LV, Camargo IX, Chiarotto GB (2021). In vivo investigation of polymer-ceramic pcl/ha and pcl/ß-tcp 3d composite scaffolds and electrical stimulation for bone regeneration. Polymers (Basel).

[7] Lee HH, Yu HS, Jang JH, Kim HW (2008). Bioactivity improvement of poly(epsilon-caprolactone) membrane with the addition of nanofibrous bioactive glass. Acta Biomater.

[8] Siddiqui N, Asawa S, Birru B, Baadhe R, Rao S (2018). PCL-based composite scaffold matrices for tissue engineering applications. Mol Biotechnol.

[9] Yan L, Li H, Xia W (2020). Bioglass could increase cell membrane fluidity with ion products to develop its bioactivity. Cell Prolif.

[10] Xie WH, Chen XY, Li YL, Miao GH, Wang G, Tian T (2020). Facile synthesis and in vitro bioactivity of radial mesoporous bioactive glass with high phosphorus and calcium content. Adv Powder Technol.

[11] Xue YM, Zhang ZJ, Niu W, Chen M, Wang M, Guo Y (2019). Enhanced physiological stability and long-term toxicity/biodegradation in vitro/in vivo of monodispersed glycerolphosphate-functionalized bioactive glass nanoparticles. Part Part Syst Char.

[12] Bachar A, Mercier C, Tricoteaux A, Leriche A, Follet C, Hampshire S (2016). Bioactive oxynitride glasses: synthesis, structure and properties. J Eur Ceram Soc.

[13] Tian T, Xie W, Gao W, Wang G, Zeng L, Miao G (2019). Micro-nano bioactive glass particles incorporated porous scaffold for promoting osteogenesis and angiogenesis in vitro. Front Chem.

[14] Raphel J, Holodniy M, Goodman SB, Heilshorn SC (2016). Multifunctional coatings to simultaneously promote osseointegration and prevent infection of orthopaedic implants. Biomaterials.

[15] Dar HY, Azam Z, Anupam R, Mondal RK, Srivastava RK (2018). Front Biosci (Landmark Ed).

[16] Chu C, Deng J, Sun X, Qu Y, Man Y (2017). Collagen Membrane and Immune Response in Guided Bone Regeneration: Recent Progress and Perspectives. Tissue Eng Part B Rev.

[17] Li X, Chen X, Miao G, Liu H, Mao C, Yuan G (2014). Synthesis of radial mesoporous bioactive glass particles to deliver osteoactivin gene. J Mater Chem B.

[18] Zhao X, Pathak JL, Huang W, Zhu C, Li Y, Guan H (2020). Metformin enhances osteogenic differentiation of stem cells from human exfoliated deciduous teeth through AMPK pathway. J Tissue Eng Regen Med.

[19] Aslanbay Guler B, Morçimen ZG, Tasdemir S, Demirel Z, Turunç E, Sendemir A (2024). Design of chemobrionic and biochemobrionic scaffolds for bone tissue engineering. Sci Rep.

[20] Ramzan F, Khalid S, Ekram S, Salim A, Frazier T, Begum S (2024). 3D bio scaffold support osteogenic differentiation of mesenchymal stem cells. Cell Biol Int.

[21] Lee J, Byun H, Madhurakkat Perikamana SK, Lee S, Shin H (2019). Current advances in immunomodulatory biomaterials for bone regeneration. Adv Healthc Mater.

[22] Wang H, Yan Y, Lan H, Wei N, Zheng Z, Wu L (2022). Notoginsenoside R1 promotes migration, adhesin, spreading, and osteogenic differentiation of human adipose tissue-derived mesenchymal stromal cells. Molecules.

[23] Li Z, Li Q, Tong K, Zhu J, Wang H, Chen B (2022). BMSC-derived exosomes promote tendon-bone healing after anterior cruciate ligament reconstruction by regulating M1/M2 macrophage polarization in rats. Stem Cell Res Ther.

[24] Toita R, Shimizu Y, Shimizu E, Deguchi T, Tsuchiya A, Kang JH (2024). Collagen patches releasing phosphatidylserine liposomes guide M1-to-M2 macrophage polarization and accelerate simultaneous bone and muscle healing. Acta Biomater.

[25] Toita R, Kang JH, Tsuchiya A (2022). Phosphatidylserine liposome multilayers mediate the M1-to-M2 macrophage polarization to enhance bone tissue regeneration. Acta Biomater.

[26] Chu C, Liu L, Rung S, Wang Y, Ma Y, Hu C (2020). Modulation of foreign body reaction and macrophage phenotypes concerning microenvironment. J Biomed Mater Res A.

[27] Zheng Z, Wang R, Lin J, Tian J, Zhou C, Li N (2022). Liquid crystal modified polylactic acid improves cytocompatibility and m2 polarization of macrophages to promote osteogenesis. Front Bioeng Biotechnol.

[28] Schlundt C, Fischer H, Bucher CH, Rendenbach C, Duda GN, Schmidt-Bleek K (2021). The multifaceted roles of macrophages in bone regeneration: a story of polarization, activation and time. Acta Biomater.

[29] Yunna C, Mengru H, Lei W, Weidong C (2020). Macrophage M1/M2 polarization. Eur J Pharmacol.

[30] Hu K, Jin Y, Chroneos Z, Han X, Liu H, Lin L (2018). Macrophage functions and regulation: roles in diseases and implications in therapeutics. J Immunol Res.

[31] Arabpour M, Saghazadeh A, Rezaei N (2021). Anti-inflammatory and M2 macrophage polarization-promoting effect of mesenchymal stem cell-derived exosomes. Int Immunopharmacol.

[32] Arabpour M, Saghazadeh A, Rezaei N (2021). Anti-inflammatory and M2 macrophage polarization-promoting effect of mesenchymal stem cell-derived exosomes. Int Immunopharmacol.

[33] Siddiqui N, Asawa S, Birru B, Baadhe R, Rao S (2018). PCL-based composite Scaffold matrices for tissue engineering applications. Mol Biotechnol.

[34] Tian T, Xie W, Gao W, Wang G, Zeng L, Miao G (2019). Micro-nano bioactive glass particles incorporated porous scaffold for promoting osteogenesis and angiogenesis in vitro. Front Chem.

[35] Li W, Liu Y, Zhang P, Tang Y, Zhou M, Jiang W (2018). Tissue-engineered bone immobilized with human adipose stem cells-derived exosomes promotes bone regeneration. ACS Appl Mater Interfaces.

[36] Wang C, Chen B, Wang W, Zhang X, Hu T, He Y (2019). Strontium released bi-lineage scaffolds with immunomodulatory properties induce a pro-regenerative environment for osteochondral regeneration. Mater Sci Eng C Mater Biol Appl.

[37] Wu S, Ma J, Liu J, Liu C, Ni S, Dai T (2022). Immunomodulation of Telmisartan-loaded PCL/PVP Scaffolds on macrophages promotes endogenous bone regeneration. ACS Appl Mater Interfaces.

